# Challenging experiences of the elderly with heart failure in the COVID-19 pandemic: a phenomenological study in Iran

**DOI:** 10.1186/s12877-023-04568-9

**Published:** 2023-12-11

**Authors:** Arash Ziapour, Javad Yoosefi Lebni, Fatemeh Mohammadkhah, Fakhreddin Chaboksavar, Parisa Janjani, Murat Yıldırım

**Affiliations:** 1 Cardiovascular Research Center, Health Institute, Imam-Ali Hospital, Kermanshah University of Medical Sciences, Kermanshah, Iran; 2 Social Determinants of Health Research Center, Lorestan University of Medical Sciences, Khorramabad, Iran; 3 Nursing Care Research Center, Health Research Institute, Babol University of Medical Sciences, Babol, I.R. of Iran; 4 Department of Psychology, Faculty of Science and Letters, Agri Ibrahim Cecen University, Ağrı, Türkiye Turkey; 5 Graduate Studies and Research, Lebanese American University, Beirut, Lebanon

**Keywords:** Challenges, Coping strategies, COVID-19, Elderly, Heart Failure

## Abstract

**Background:**

Patients with heart failure (HF), especially the elderly, faced many challenges during the COVID-19 pandemic, which need to be explored. The present study aimed to analyse the challenging experiences facing the elderly with heart failure during the pandemic in Iran. To achieve this aim, a qualitative approach to research was employed.

**Methods:**

The present qualitative research employed a phenomenological approach to study 12 elderly with heart failure visiting Imam Ali Hospital during the COVID-19 pandemic in Kermanshah, Iran. The data collection occurred between August 5, 2022, and November 21, 2022. The participants were selected through a purposive sampling method and interviewed using a semi-structured interview. MAXQDA10 software was used for data organisation and the Colizzi analytical technique for data analysis. Guba and Lincoln’s criteria were also used to evaluate the trustworthiness of the qualitative research.

**Results:**

A total of two categories, 8 subcategories, and 110 primary codes were extracted from the data. The two categories were challenges and coping strategies. The former included the sub-categories of economic issues, fear, anxiety, and fear of death; limited access to health care; quarantine issues; disruption of family life; and medication non-adherence. The subcategories of the latter were faith in God, social support, and self-care behaviors.

**Conclusions:**

The present study revealed the challenges experienced and the coping strategies employed by elderly patients with heart failure during the pandemic in Iran. A knowledge of these challenges and experiences during the COVID-19 pandemic can raise healthcare workers’ awareness of the elderly patients’ strategies used to cope with the virus.

**Supplementary Information:**

The online version contains supplementary material available at 10.1186/s12877-023-04568-9.

## Background

The mortality rate associated with COVID-19 is increasing [1]. As predicted by the World Health Organization (WHO), a further spread of the disease will become the third cause of mortality in the world by 2030 [2, 3]. During the pandemic, though the virus is a risk factor for all age groups, it has been well-proven that the prevalence is higher in the elderly. In fact, the number of elderly patients with COVID-19 is higher than that of other age groups [4]. Liu et al. studied 4,021 positive cases of COVID-19 and reported a mortality rate of 5.3% in the elderly population (≥ 60 years) and a rate of 1.4% in the young and middle-aged [5]. Based on the existing epidemiological data, older patients with SARS-CoV-2 showed a higher risk of mortality than younger patients [6, 7]. In agreement with these findings, a large-scale study with 4,021 participants infected with COVID-19 showed that patients over 60 years of age had a significantly higher mortality rate than those under 60 years of age [8].

Iran will face a large number of elderly population in the near future [9]. Iran’s elderly population has increased from 6.6% in 1996 to 9.3% in 2016, and the number of elderly people is expected to double between 2015 and 2030 [10]. Scientific evidence shows that as people age, the incidence and prevalence of cardiovascular diseases increases, so that in people over 65 years old, the prevalence and incidence of this disease is 10 out of every 1000 people [11].

The elderly population is anticipated to increase from 800 million in the world to two billion by 2050, of which 23% belong to developed countries and 9% to developing countries [12, 13]. Therefore, it is worth investigating the issues related to the elderly population. Besides the COVID-19 pandemic and the critical conditions facing the elderly, facts and figures show that out of every ten mortality cases induced by the virus, eight are associated with comorbidities such as cardiovascular diseases, hypertension, and diabetes [14]. Although COVID-19 was initially considered a respiratory disease, in the actual pandemic context, it quickly became evident that the heart is a primary organ threatened by the virus, and patients infected with the virus may develop a heart disease too [1, 13, 15–19].

Chronic heart failure (HF) is a complex clinical syndrome and a progressive and debilitating chronic disorder marked by shortness of breath, fatigue, etc., and is considered a major threat to public health [20]. The relationship between COVID-19 and HF is complex and two-way. A history of HF is a risk factor for experiencing and transmitting more severe cases of COVID-19. HF can result from myocardial damage caused by COVID-19. Research findings also confirm this two-way relationship. For example, after the emergence of the coronavirus, early reports in China showed that cardiovascular diseases, such as the HF, were among the common comorbidities in patients with COVID-19 [4].

The recurrence or exacerbation of HF is a common complication in patients with COVID-19, and the mortality rate has been reported to be as high as 36% [6]. In a seminal study conducted on 799 patients in Wuhan, HF was one of the most common complications of COVID-19. HF was found to prevail in 24% of all patients and led to death in 49% of patients [21]. In another study, HF was diagnosed in 23% of all patients, 52% of whom died [6]. Despite the importance of this two-way relationship and the need to pay close attention to it, there is further research evidence to show that patients with heart problems have faced different challenges to receive services [22].

Most doctors admitted that the number of patients visiting for treatment and medical care decreased during the pandemic, which raises concerns for significant side effects in many cardiovascular patients, especially those with HF [23]. Koning et al. showed that people cancel or postpone medical treatments for a fear of the virus. As some symptoms of the heart disease are similar to those of COVID-19, the diagnosis becomes more difficult; thus, the quality of services is lowered [24]. If patients fail to visit a health centre as soon as possible, their health is seriously threatened [25]. Non-adherence to medication increases the mortality rate induced by heart diseases due to delayed medical care provision compared to the pre-pandemic era [26].

There is research evidence that heart diseases and COVID-19 mostly damage the elderly compared to any other age group [6, 7]. The elderly are prioritised in quarantine due to their physical condition and greater vulnerability to the virus [27]. Quarantine and lock-in rules made some elderly citizens feel confined and imprisoned [28, 29]. In many daily tasks and activities, they felt more reliant on others than ever before [30]. Evidently, the higher susceptibility of the elderly led to their higher perceived anxiety and stress [30] and a fear of disease and death [21]. A review of the related literature shows the challenges facing the elderly during the COVID-19 pandemic. Though a few quantitative studies have explored HF and the incidence of COVID-19 in different populations [31–33], almost none have examined the lived experience of patients with HF, especially the elderly population. Therefore, raising awareness of the challenges and coping strategies of patients with HF during the COVID-19 infection can lead to the adoption of effective measures. Since qualitative phenomenological studies are useful for making sense of experiences personally perceived and interpreted [34], the present study aimed to explore the experiences and challenges of the elderly with HF during the COVID-19 pandemic using a qualitative approach. The study was conducted in Iran. There are hopes that the present findings contribute to the development of an interventional programme and promote and support comprehensive recovery (i.e., physical, psychological, and social) among elderly patients with COVID-19.

## Methods

### Design of study and participants

This research employed a qualitative approach and a phenomenological method. Phenomenology relies on philosophical assumptions to study the lives or lived experiences of people [35]. Phenomenology sees the world as the person lives it and tries to discover the meanings that s/he makes in daily life. It helps make new meanings and set aside the experiences to achieve a new understanding of life in the world [36]. The research population in the present study includes the elderly with HF visiting Imam Ali Hospital during the COVID-19 pandemic in Kermanshah, Iran. The inclusion criteria were a diagnosis of HF by a cardiologist, a minimum age of 60 years, a visit to Imam Ali Hospital in Kermanshah for healthcare services, a willingness to participate in the study, and consent to audio-recording the interview. The exclusion criteria were having a severe mental disorder, not being able to speak, and failing to complete the interview.

A purposeful sample was used to select the participants. Participants were selected who had more information and could better provide their experiences to the researcher. It means that they have experienced more problems and challenges during the Covid-19 era. Initially, the participants were found among those visiting Imam Ali Hospital in Kermanshah, a hospital for cardiac patients. The patients were invited to participate in the research after the researchers explained the objectives and procedure of the study. A semi-structured interview was held with the participants at a time and place of their convenience. Most interviews were conducted at the Heart and Vascular Research Center of Kermanshah University of Medical Sciences. At first, the interviewer introduced himself and the purpose of the study. As a warm-up, the interviews began with questions about the experience of the first days after the publication of the news on COVID-19 and continued with a general question(Table 1).

**Table 1:** Interview guide

• Can you tell me about your experience with HF during COVID-19?
• What were the most important challenges and problems you faced (health, treatment, personal matters, family, etc.) during the pandemic concerning your HF? Please explain.
• What health and treatment services were provided to you during the pandemic, and how did you receive the services? Please explain.
• Concerning the problems you faced during the pandemic, how did you react, i.e., how did you cope with the situation?
• What do you think can help you better face this critical condition?
• How do you advise other people with HF to better cope with this condition?

While listening to the interviewees, recording their voices, and analysing the content, certain other points were also raised for further discussion. Each interview lasted for 45–63 min. Most interviews were conducted in the morning and afternoon. All interviews were recorded with the participants’ consent. The interviews were conducted by the fifth author of the article, (PJ), who has a Ph.D. in psychology and is also proficient in qualitative research and the principles of interviewing. She assisted a cardiologist and an echocardiography specialist, who were also attending the interview session. The role of this person was that, if the participants used medical terms in the interview and these corrections or sentences were not understandable to the researcher, he would explain them. They also provided additional comments about the patient’s condition to the researcher. After a patient was examined by the cardiologist-echocardiography specialist, s/he was interviewed by the (PJ), who has a Ph.D. in psychology. The interviewer attempted to create a sympathetic and friendly atmosphere where the interviewee could openly and willingly share his or her experiences of challenges faced during the pandemic. The data collection lasted from August 5, 2022, to November 21, 2022. Interviews continued until data saturation, which reached saturation with 12 participants.

### Data analysis

Data analysis was done at the same time as the data collection, and the Colizzi method was employed [37]. The data analysis started after the first interview and it was not the case that the analysis and coding started after the end of the 12 interviews. The codes that were created were asked in the form of questions in the subsequent interviews so that the codes could be constantly reviewed and compared. Thus, after each interview, the first researcher (AZ) and his colleague(JYL) listened to the content of the interview twice and then transcribed the conversation and transferred it into the MAXQDA10 software. The researchers (AZ & JYL) reviewed the transcripts several times to make sense of the participants’ feelings and experiences. Then they did the data coding and analysis (AZ & JYL). First, the primary coding was done by exploring and forming the main themes. These themes were then further organised into several subcategories based on similarities and differences. Finally, they were organised into major categories in terms of the consistency between and among the subcategories. The emergent codes were transferred into the MAXQDA 10 software. The software helped in the categorization of codes.

### Trustworthiness

To assess the quality of the findings, Guba and Lincoln’s trustworthiness criteria were used [38]. To assure the credibility of the data, the researchers’ long-term engagement in the field was maintained. The place of work of most of the authors of the article was in the Heart and Vascular Research Center of Kermanshah University of Medical Sciences, which was located inside the hospital. Therefore, in the midst of Covid-19, they were constantly faced with heart patients and their problems and could better understand their words. At the end of each interview, the interviewer summarised his interpretation of the participants’ accounts generally to ask for their approval. Also The research findings section was sent to some participants via email and was approved by them. The present researchers tried to include a sample with the maximum variety of demographic features. The confirmability of findings was assured by maximising the researcher’s impartiality and agreement on codes, categories, and subcategories by all members of the research team as well as experts in qualitative research and cardiac issues. The findings section of the study was sent to people specializing in qualitative research, as well as people specializing in cardiovascular disease. They were asked to comment on whether the process of research and data analysis was done correctly. And whether this study was able to do it or not. which was approved by them. Also, the raw data and all interviews file and transcripts were kept for later revisions. To substantiate the dependability of findings in the data coding and analysis, the naming of categories and subcategories was done with all authors’ agreement. To assess the transferability of the findings, while analysing the findings, rich descriptions of the conditions were given. For each category and subcategory, there were many direct quotations from the participants’ accounts.

## Results

The present participants were 12 in number (8 male and 4 female patients). Their ages ranged between 60 and 83 years, and their levels of education ranged from elementary to bachelor’s degrees (Table 2). Also, 2 categories, 8 subcategories, and 110 primary codes were extracted from the analysis (Table 3).

**Table 2:** Participants’ demographic information

Variable	Group	f.	%
Sex	Male	8	66.7
	Female	4	33.3
Age	60–70	9	0.75
	71–80	2	16.7
	80>	1	8.3
Marital status	Single	1	8.3
	Married	11	91.7
Education	<diploma	5	41.7
	Diploma	4	33.3
	Associate degree	2	16.7
	Bachelor’s degree	1	8.3
Occupation	Disabled	1	8.3
	Retired	4	33.3
	Freelancer	5	41.7
	Housewife	1	8.3
	Employed by government	1	8.3

**Table 3:** Categories and subcategories extracted from interviews with patients in COVID-19 pandemic

Categories	Subcategories
Challenges	Economic issues
	Fear and Anxiety
	Limited access to health services
	Quarantine (lockdown) issues
	Non-adherence to medication
Coping strategies	faith in God
	Social support
	Self-care behaviors

### Challenges

The first category that came out of the experiences of the participants was the problems that older people with HF had during the COVID-19 pandemic. There were problems with money, fear, anxiety, or fear of death; limited access to health care; problems with quarantine; problems with family life; and not taking medications as prescribed.

### Economic issues

With the spread of COVID-19, many businesses were down. Yet, providing the supplies needed for infection prevention, such as alcohol and masks, increased the cost of living. People with HF were expected to follow health protocols more closely than others due to their high-risk condition. Thus, their costs of living suddenly increased significantly more than before, which was troublesome. Also, due to the high medical costs, some participants preferred not to visit health centres as frequently as before, and this could threaten their health. Here are some relevant excerpts:

Participant #1 (male, 63 years old): “Honestly, it was a painful experience—the attack of an unknown disease that had no cure, the associated economic problems, the lack of motivation, and the costs imposed for disinfection and healthcare.”

Participant #2 (male, 60 years old): “I found it hard to buy the drugs I needed because they were expensive.”

Participant #7 (female, 66 years old): “It was very difficult for us. On the one hand, my husband and son were unemployed; on the other hand, the Corona virus had made things hard with the increased costs of health care. I could not visit a doctor as the costs of visits and treatment were high”.

### Anxiety and fear

Another subcategory of challenges extracted from the interviews was anxiety and fear. People were scared because more and more people were getting sick with the virus and dying as a result. High-risk groups, such as HF patients, who were more exposed to the harm COVID-19 caused, expressed this feeling more strongly. Most participants felt extreme stress and fear and were worried about the future and what could happen to them if they got infected with the virus. Patients with HF perceived themselves as being near death as they were at a higher risk of COVID-19 infection, accounting for a high rate of mortality. This issue increased their anxiety about death, so most participants were expecting death.

Participant #4 (female, 61 years old): “The fear of the virus, especially for those with a comorbidity, was very terrible.”

Participant #5 (male, 75 years old): “Considering my age and the open-heart surgery I had, I was tremendously scared.” Even if I did not have a heart problem, the COVID-19 infection scared me. I had the fear of dying alone, not seeing my children and grandchildren around”.

Participant #8 Participant #2 (male, 60 years old): “Since I realised I had HF, my first impression was that my life was over and I could expect any moment. It really hurts when you feel hopeless, waiting for a stroke or death.”

Participant #3 (male, 67 years old): “I was desperately thinking my heart disease could be associated with the COVID-19 and that I would die soon. I think the anxiety and stress that the thought of death causes is stronger than death itself.”

Participant #4 (male, 61 years old): “The main challenge for me was the internal conflict and disturbing thoughts I developed. I was scared of death and worried I could have a stroke and die from the heart disease.”

### Limited access to health care services

With the emergence of COVID-19 in Iran, the lack of health and medical equipment, both in hospitals and public pharmacies in cities, created problems for many families. Considering that heart patients need to visit pharmacies and medical centers continuously. The lack of medical supplies and medicine as well as the overcrowding of pharmacies caused them to be under more pressure. Also, due to the crowdedness of the hospitals, doctors were less able to spend time with heart patients and visit them. Therefore, the problems of heart patients were more than before due to limited access to medical and health services.

Participant #1 (male, 63 years old): “It was a little difficult to get the drug at first.”

Participant #3 (male, 67 years old): “Sometimes I was wondering what to do if the medicine was out of stock.”

Participant #4 (61 years old): “At the beginning of the pandemic, there was an awful psychological climate due to the lack of detergents, masks, gloves, etc. In my opinion, the biggest problem was attending medical centers, the fear of being infected or carrying the virus and the restrictions on traffic. The rumour spread that medicine would soon be out of stock”.

Participant #5 (male, 75 years old): “My biggest problem was the availability of healthcare services, and this issue made me truly anxious. At heart, I felt worried and sometimes afraid of the limitations and lack of medicine, especially when the pandemic was in the worst condition. I was a patient with HF who had had open-heart surgery”.

### Quarantine issues

Considering that patients with HF were among the high-risk groups for transmitting the COVID-19 virus and the mortalities, it was recommended that they observe the quarantine conditions so as not to get infected with the virus. Compliance with the quarantine conditions put a lot of pressure on patients because it separated them from family and friends and posed many problems such as loneliness, despair, isolation, aloofness, boredom, and uncertainty about the quarantine time.

Participant #1 (male, 63 years old): “We did not have a particular family problem, except that all family members were worried, and this made us nervous and aggressive at the beginning of the pandemic as we spent more time together, especially during the quarantine.”

Participant #11 (female, 60 years old): “I really felt lonely. No one visited me. I did not know how long this quarantine would last, and it was bothering.”

Participant #9 (male, 79 years old): I missed all the freedom I had before the pandemic. It bothered me not to be able to go out, see friends and colleagues, go to a park, play chess, touch everything you want, and the like”.

Participant #1 (male, 63 years old): “I had a hard time. I was disappointed; I could not tolerate anyone.”

### Non-adherence to medication

According to the illness of the participants, most of them needed to take medicine continuously or be under the supervision of a doctor and refer to doctors and medical centers so that they do not have any special problems. But due to the conditions of Corona and the restrictions after that, most of them delayed taking medicines or going to the doctor or did it irregularly, which could threaten their health more than before and even lead to to their death.

Participant #8 (female, 70 years old): “Very often, I failed to follow the doctor’s advice. It was hard to get the drugs. Also, for fear of infection, I was afraid of visiting a specialist. Sometimes, when I ran out of drugs, I simply did not take them for a couple of days because I was afraid of leaving home and purchasing the drugs”.

Participant #10 (male, 68 years old): “From the very beginning, I failed to adhere to my medication. Things got worse, and I still preferred not to leave home to see a doctor for an examination, an echocardiogram, and so on. There were times I ran out of medicine for days, but nothing serious happened”.

### Coping strategies

The second category extracted from the participants’ accounts was coping strategies. Many participants lowered the severity of their condition and facilitated their recovery through certain behaviours and strategies, such as having a faith in God, seeking social support, and showing self-care behaviors, which made them feel more capable of coping with the conditions caused by the COVID-19 pandemic.

### Faith in god

Another strategy used by the participants to cope with the pandemic crisis was their faith in God. In fact, the participants tried to maintain peace by communicating more with God through prayers. Some others considered the spread of the disease a kind of divine act that put their minds at peace”.

Participant #2 (male, 63 years old): “Faith in God and the fact that there was someone who cared for me gave me hope. As my late mother said, nothing happened unless God wanted it to happen”.

Participant #5 (75-year-old man): “I think, in any critical condition, remembering God and trusting Him in the first place is the panacea. Faith in God is the best we can do. In miseries, we can appeal to Him and find peace in His company”.

Participant #8 (female, 70 years old): “I appealed to the innocent imams and God. Whenever I was upset or worried, I spoke to God and asked Him to protect me”.

### Social support

Another strategy the participants employed during the pandemic was to get social support. Some liked their family members to be with them for more support, and when face to face meeting was not possible, they tried to receive this support via the cyber space”.

Participant #2 (male, 60 years old): “What further annoyed me was that I lost the support of those around me. No one came along because of the virus, and all my friends and family were just calling me on phone, while I preferred to see them face to face, and have a friendly chat”.

Participant #3 (male, 67 years old): “More than ever before, I wanted all my family members around. Yet, because of the virus, it was not possible. I tried to talk to them more on the phone. My family was more attentive to me and encouraged me, which could calm me down.”

Participant #4 (female, 61 years old): “I think in any critical condition, relatives, friends, and beloved ones can be a great help. These are people to whom we are emotionally linked and, thus, can help a lot”.

### Self-care behaviours

Some participants tried to take good care of their health as much as they could to cope with the challenges during the COVID-19 pandemic. Some tried to protect themselves against the disease by adhering to a nutritious diet, and others did so by doing exercises at home and observing more health protocols.

Participant #1 (male, 60 years old): “I tried to eat as much healthy food as I could, so as to strengthen my body and not die of the COVID-19.”

Participant #7 (female, 66 years old): “I appealed more to traditional medicine to keep fit. Then, I followed traditional medicine more closely so that no problem would occur to me. I was also very careful not to catch a cold or smoke.”

Participant #12 (female, 64 years old): “I did everything to protect myself from the COVID-19 infection. I followed all the health protocols that were compulsory so as not to get infected with the virus.”

Participant #4 (female, 61 years old): “To keep fit, I try to exercise a little more than before and not to be inactive at home.”

## Discussion

The present study explored the challenges faced by the elderly with HF during the COVID-19 pandemic using in-depth interviews with a sample of patients with HF. The design of the study was phenomenological. This research was carried out to analyse the patients’ experiences and challenges during the COVID-19 pandemic using a qualitative approach.

A major challenge facing the patients with HF was the economic situation. In addition to the loss of life, the COVID-19 pandemic had irreparable economic effects [39]. A lack of financial support for the elderly with heart diseases is associated with an increased risk of ischemic heart disease and stroke [40]. Aging and chronic diseases such as metabolic, respiratory, and cardiovascular problems, together with the COVID-19 pandemic, challenged the economic and health systems of the world [41]. Therefore, government and public support is suggested for the economic support of these patients.

Among the challenges experienced by the participants was their perceived fear. With the rapid spread of the pandemic, the elderly with chronic diseases such as heart disease felt a great fear [42]. which is consistent with the results of Jamili et al. and Chee et al. studies [38, 40].

Social media can act effectively in raising public awareness [43]. The pandemic, with its rapid increase in infection and subsequent mortality, led to different degrees of anxiety, fear, mistrust, and rumour spreading among the public. Therefore, a rapid dissemination of scientific information can be a suitable and effective way to reduce public fear and anxiety caused by a new epidemic or any critical condition [44].

Another challenge the participants mentioned in the present study was the anxiety and fear, which is a complex issue that includes the fear of one’s own death and that of others [45]. The threat of COVID-19 infection and death and the unsympathetic form of burial and mourning, which was contrary to expectations and preferences, were among the factors causing anxiety for the elderly [46]. Anxiety about death refers to the fear and harassment caused by the awareness of one’s own and others’ deaths, which causes fear and worry from within [47, 48]. The further spread of the virus and everyday news added to the severity of this issue [44]. Evidently, the best solution to dispelling false rumours about the disease is to equip people with both health literacy and media literacy [44] so that they can get rid of their anxiety and fear of death.

Another challenge extracted from the participants’ accounts in the present study was limited access to health care services which is consistent with the results of other studies [29, 49–55].

The pandemic affected the health sector in many ways. The limited human resources, intensive care beds, and medical equipment were increased in places with an ever-growing population of COVID-19 patients. Daily care services such as health checkups, cancer screenings, or vaccinations decreased. There was also less primary care provided by general practitioners or specialists [49], which seems to have limited access to health care for the elderly with a heart disease.

Among the other challenges the participants pinpointed in this study were the quarantine issues. During the COVD-19 pandemic, it was imperative that the elderly be isolated and keep a social distance. Quarantine and social isolation in critical conditions were sources of anxiety and other psychological problems for this population. Health professionals should be aware of these issues and attempt to take measures to reduce the adverse effects of quarantine and social isolation [50] which is consistent with the results of other studies [53–56].

Although many countries were taking measures to slow down the spread of the disease, the frustration induced by the quarantine was inevitable as people fell behind in their daily activities. The social and physical contacts were limited. This condition could also increase the level of depression, anxiety, post-traumatic stress disorder, and socio-economic distress [51].

To increase people’s maintenance of stay-at-home orders, it is suggested to provide more economic support and education and also raise public awareness of the quarantine [53]. Other interventions can help increase interactions with important people, family, and close friends; effectively use the internet system; develop and maintain social support networks; and set up voluntary projects and organisations in society to prevent the adverse psychological effects of quarantine on the elderly [33].

The availability of smartphones and high-speed internet connections during the pandemic lockdown could introduce a dramatic change to cardiac rehabilitation programmes using telemedicine services to improve the health condition of more patients with HF [33].

Another challenge mentioned by the participants was non-adherence to medication which is consistent with the results of other studies [51, 57, 58].

It seems that the CDC guidelines, the fear of infection or death, and stay-at-home orders significantly reduced the visits to doctors and adherence to medication. Perhaps the most effective type of health care is remote services, available in many parts of the world and often known as telemedicine [52].

One strategy mentioned by the participants to cope with the disease was faith in God which is consistent with the results of other studies [41, 53, 59].

In this case, nurses and other medical staff with a stronger faith in God and religious beliefs can more effectively help patients with heart disease improve their health [53].

Another strategy to cope with the disease was to receive social support which is consistent with the results of other studies [medication which is consistent [60, 61].

Among the strategies to cope with the disease mentioned by the participants were self-care behaviors. As the individual participants commented, self-care behaviours were significantly affected during the pandemic [54, 55] which is consistent with the results of other studies [62, 63].

In their study, Valizadeh et al. argued that one solution to the challenges of the pandemic was to show self-care behaviour and maintain personal health [56]. The fear of infection can influence people’s self-care behaviours [54]; During the COVID-19 pandemic, telephone-delivered self-care education for patients with HF can improve self-care behaviours in patients with heart disease [64]. Telephone-delivered education can be used as an effective intervention to promote self-care behaviours in the elderly with cardiovascular diseases.

This study showed that the experiences of the elderly with HF during the pandemic were unique. As the lived experience showed, what is represented in their perceptual world can include economic issues and concerns, fear, anxiety, and fear of death; limited access to health care services; quarantine issues; disrupted family life; and medication non-adherence, all of which can somehow disrupt the treatment process.

### Strengths and limitations

The present research is among the few existing studies that investigated the lived experiences and challenges of patients with HF during the COVID-19 pandemic. The findings can provide insights for policymakers, psychologists, social workers, and public health officials in finding practical solutions to these problems. Nevertheless, this study, like many qualitative studies of COVID-19, faced a number of limitations. One limitation of the present study is that due to the fact that elderly heart patients are not in good physical condition and have relatively weaker cognitive performance, they may have faced problems in understanding and understanding the questions correctly, and additional explanations were tried to solve this limitation. Other limitations are the small sample size and the cultural context of the study, which can lower the generalizability of findings.

## Conclusion

The results showed that The experiences of patients with HF during the COVID-19 pandemic showed this disease affected all aspects of their health (e.g., physical, mental, economic, familial, and social). Therefore, understanding patients’ challenges, experiences, and concerns about infection can help healthcare workers understand and be aware of its consequences. During the quarantine period, psychiatrists and psychologists played an important role in helping people overcome the psychological consequences of the disease (e.g., stress and depression) for these patients. Providing telemedicine and telephone-delivered counselling services for people, educating patients, and providing medicine are among the measures that can help maintain the continued healthcare of patients with a heart disease. It is suggested in situations such as possible future crises and similar epidemics; Officials and politicians take measures to financially support heart patients in the form of insurance coverage and have free medical services. Also, educational interventions in order to improve the mental and spiritual health and self-care activities of heart patients and family-oriented trainings in order to improve social support are necessary.

### Electronic supplementary material

Below is the link to the electronic supplementary material.

Supplementary Material 1

## Data Availability

The datasets used in the study are available from the corresponding author on reasonable request.

## Abbreviations

CE: Challenging Experiences
EWHF: Elderly with Heart Failure
CP: Covid-19 Pandemic
PS: Phenomenological Study
HF: Heart Failure
