# Supplementary material for: Challenging experiences of the elderly with heart failure in the COVID-19 pandemic: a phenomenological study in Iran
Source: BMC Geriatr. 2023 Dec 11;23:834. doi: 10.1186/s12877-023-04568-9 (PMC10712086; doi:10.1186/s12877-023-04568-9)
Supplement: Supplementary file 1 — Supplementary Material 1 [file 12877_2023_4568_MOESM1_ESM.pdf]

Consolidated criteria for reporting qualitative studies (COREQ): 32-item checklist

NO	Item	Guide
1	Interviewer/facilitator	in Methods(paragraphs 4) in The first author of the study, who is a doctor of Psychology and a qualitative research specialist.
2	Credentials	**Author Details**
3	Occupation	PhD Psychology
4	Gender	Female
5	Experience and training	The researchers were well acquainted with qualitative research and interviewing and had sufficient expertise in this field.
6	Relationship established	To ensure a comfortable atmosphere, the researcher offered a brief summary of his or her personal and educational background at the start of the interview, then emphasized the importance of the research and how to conduct and report it.
7	Participant knowledge of the interviewer	To ensure a comfortable atmosphere, the researcher offered a brief summary of his or her personal and educational background at the start of the interview, then emphasized the importance of the research and how to conduct and report it.
8	Interviewer characteristics	Methods (paragraphs4)
9	Methodological orientation and Theory	Methods (paragraphs1) Phenomenological method
10	Sampling	Methods (paragraphs2) Purposive sampling
11	Method of approach	Methods (paragraphs1)
12	Sample size	12 Participants
13	Non-participation	N/A
14	Setting of data collection	Most interviews were conducted at the Heart and Vascular Research Center of Kermanshah University of Medical Sciences.
15	Presence of non-participants	Methods (paragraphs3)
16	Description of sample	Yas in Findings, Table 2
17	Interview guide	Yas in Methods, Table 1
18	Repeat interviews	NO
19	Audio/visual recording	Yas In this study, all interviews were recorded..
20	Field notes	N/A
21	Duration	Methods (paragraphs3)
22	Data saturation	Methods (paragraphs3) In this study, the interviews continued until saturation (12 Participants)
23	Transcripts returned	Methods (**Trustworthiness**)
24	Number of data coders	2 categories, 8 subcategories, and 110 primary codes were extracted from the analysis (Table 3).
25	Description of the coding tree	N/A
26	Derivation of themes	Table 3 and Results
27	Software	MAXQDA software
28	Participant checking	Yas in Methods**, Trustworthiness**

29	Quotations presented	Yas in Results, All paragraphs
30	Data and findings consistent	Results , All paragraphs
31	Clarity of major themes	2themes , Table3
32	Clarity of minor themes	8 subcategories, and, Table 3
